# Pathophysiology of Doxorubicin-Mediated Cardiotoxicity

**DOI:** 10.3390/toxics13040277

**Published:** 2025-04-05

**Authors:** Roberto Arrigoni, Emilio Jirillo, Carlo Caiati

**Affiliations:** 1 Institute of Biomembranes, Bioenergetics and Molecular Biotechnologies (IBIOM), National Research Council, 70124 Bari, Italy; 2 Interdisciplinary Department of Medicine, Section of Microbiology and Virology, School of Medicine, University of Bari “Aldo Moro”, 70124 Bari, Italy; emilio.jirillo@uniba.it; 3 Unit of Cardiovascular Diseases, Department of Interdisciplinary Medicine, University of Bari “Aldo Moro”, 70124 Bari, Italy; carlo.caiati@uniba.it

**Keywords:** cardiotoxicity, doxorubicin, ferroptosis, inflammation, oxidative stress, sirtuins

## Abstract

Doxorubicin (DOX) is used for the treatment of various malignancies, including leukemias, lymphomas, sarcomas, and bladder, breast, and gynecological cancers in adults, adolescents, and children. However, DOX causes severe side effects in patients, such as cardiotoxicity, which encompasses heart failure, arrhythmia, and myocardial infarction. DOX-induced cardiotoxicity (DIC) is based on the combination of nuclear-mediated cardiomyocyte death and mitochondrial-mediated death. Oxidative stress, altered autophagy, inflammation, and apoptosis/ferroptosis represent the main pathogenetic mechanisms responsible for DIC. In addition, in vitro and in vivo models of DIC sirtuins (SIRT), and especially, SIRT 1 are reduced, and this event contributes to cardiac damage. In fact, SIRT 1 inhibits reactive oxygen species and NF-kB activation, thus improving myocardial oxidative stress and cardiac remodeling. Therefore, the recovery of SIRT 1 during DIC may represent a therapeutic strategy to limit DIC progression. Natural products, i.e., polyphenols, as well as nano formulations of DOX and iron chelators, are other potential compounds experimented with in models of DIC. At present, few clinical trials are available to confirm the efficacy of these products in DIC. The aim of this review is the description of the pathophysiology of DIC as well as potential drug targets to alleviate DIC.

## 1. Introduction

Anthracyclines [doxorubicin (DOX), daunorubicin, epirubicin, and idarubicin] are drugs utilized for their effects on cancer, but their use is limited due to cardiotoxicity [1]. The reduction of the left ventricular ejection fraction represents the most severe side effect, which occurs in 9% of patients undergoing treatment for anthracyclines. The other cardiotoxic symptoms encompass congestive heart failure, arrhythmia, and myocardial infarction.

Anthracyclines are used to treat various malignancies, including leukemias, lymphomas, sarcomas, and bladder, breast and gynecological cancers in adults, adolescents, and children [2]. DOX-induced cardiotoxicity (DIC) has been intensively investigated. The cumulative dose represents a major risk of DIC: patients receiving >400 mg/m^2^ DOX cumulative doses are at very high risk. In particular, the incidence of cardiomyopathy is reported to be 5% at a cumulative dose of 400 mg/m^2^, 26% at 550 mg/m^2^, and 48% at 700 mg/m^2^ [3,4]. However, subclinical cardiac dysfunction can be observed at lower cumulative exposures, up to a mean cumulative dose of 240 mg/m^2^, with a range of 50 to 375 mg/m^2^ [5]. Among the side effects, it is possible to distinguish between acute and chronic DIC. Acute DIC shares many similarities with acute myocarditis; in particular, acute doxorubicin cardiotoxicity is considered a rare clinical event, although several studies indicate that it is more common than previously thought (11–21%) and predicts unfavorable outcomes [6]; on the other hand, chronic DIC can appear after months or years from the initial treatment, being characterized by dilated cardiomyopathy and the irreversible reduction of the left ejection fraction, with eccentric ventricular hypertrophy and increased cardiac mass, often associated with the clinical syndrome of heart failure [7,8].

A position paper elaborated by the European Society of Cardiology reported that among a cohort of cancer survivors, one-third of deaths could be attributed to chronic cardiotoxicity [9]. Another report has documented that 20% of cancer survivors manifest left ventricular dysfunction, with a larger proportion in children [10].

Hence, it is imperative to monitor the left ventricular function in patients on DOX, before and after the completion of anthracycline-based chemotherapy, to detect early myocardial damage [11]. In this regard, echocardiography is the more suitable technique since it does not involve exposure to damaging ionizing radiation [12]. With this technique, not only should the systolic function be assessed and monitored but also the diastolic left ventricular function, with the diastolic Doppler parameters of the left ventricular function being even more sensitive than the standard ejection fraction in revealing early myocardial damage [13,14]; also, cardiovascular biomarkers have to be assessed and monitored [15].

Several risk factors predispose patients to DIC, such as hypertension, hyperglycemia, and dyslipidemia [16]. In particular, glycemic and lipidic disorders can increase the levels of fatty acids and cytokines, with accumulation of fat droplets in the myocardium [17]. Other risk factors include the extremes of age, female gender, prior mediastinal radiation therapy, concomitant treatment with cyclophosphamide, trastuzumab or paclitaxel, and the presence of cardiac disease [18]. There is evidence that DOX exerts its anticancer activity through two major mechanisms: (1) inhibiting DNA and RNA synthesis in dividing cells by stopping the replication and transcription processes, and (2) generating iron-mediated free radicals, causing cell damage to cell membranes, proteins, and DNA [19,20,21].

DIC’s etiology is multifactorial, being based on a combination of nuclear-mediated cardiomyocyte death and mitochondrial-mediated death [22,23]. Moreover, cardiomyocytes exhibit low levels of antioxidant protection, being more susceptible to free radicals and reactive oxygen species (ROS) damage [24].

The aim of the present review will be the description of the major mechanisms leading to DIC. A better understanding of the cellular and molecular events implicated in the pathophysiology of DIC may help in searching for potential treatments for DIC prevention or cardiotoxicity reduction.

## 2. Major Pathogenetic Mechanisms of DIC

DOX toxicity is evident in many organs, such as the brain, liver, cardiac muscle, and skeletal muscle [25]. According to recent research, doxorubicin exerts its anticancer effects through multiple mechanisms; DIC is correlated to DNA instability, oxidative stress, altered autophagy, inflammation, and apoptosis.

### 2.1. DNA Instability and Topoisomerase Inhibition

**Figure 1:**
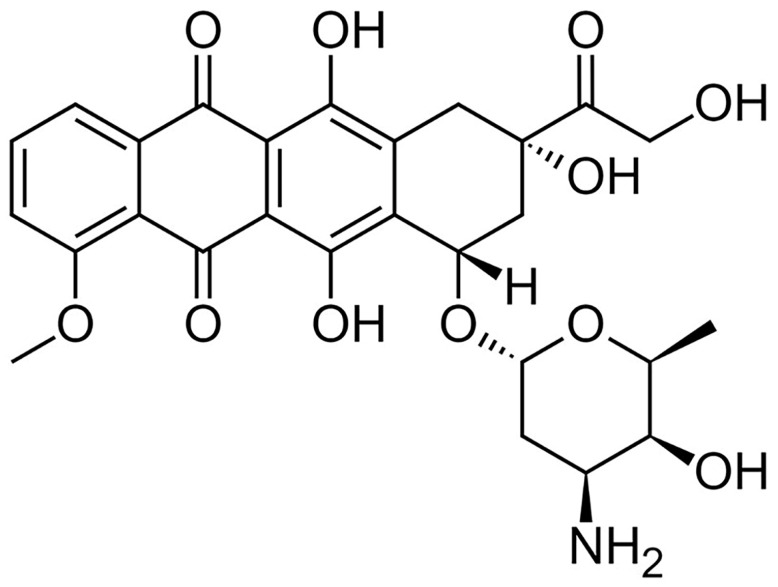
Doxorubicin’s chemical structure.

Doxorubicin can display its cytotoxic effects by disrupting DNA stability by directly intercalating into the DNA structure (see Figure 1) [26]. The drug’s planar anthracycline structure allows it to intercalate between DNA base pairs, forming a covalent bond with guanine on one strand and hydrogen bonds on the opposite strand, causing torsional strain and physical obstruction of essential processes such as replication and transcription. This intercalation not only distorts the DNA helix but also generates reactive oxygen species (ROS) through redox cycling, further exacerbating the DNA damage via oxidative base modifications and strand breaks. The resulting genomic instability activates the DNA damage response pathways, ultimately leading to cell cycle arrest and apoptosis in rapidly dividing cancer cells.

A critical aspect of doxorubicin’s mechanism is its inhibition of topoisomerase II, an enzyme essential for managing DNA supercoiling during replication and transcription [27]. By stabilizing the topoisomerase II–DNA cleavage complex, doxorubicin prevents the enzyme from religating severed DNA strands, converting transient topoisomerase-II-induced breaks into persistent double-strand breaks (DSBs) [28]. These DSBs overwhelm the cell’s repair capacity, particularly in highly proliferative tumor cells, triggering p53-mediated apoptosis. However, this same mechanism contributes to the drug’s dose-limiting cardiotoxicity, as cardiomyocytes, which have limited regenerative capacity, accumulate irreversible DNA damage and mitochondrial dysfunction due to ROS generation.

Recently, it was demonstrated that doxorubicin can also alter RNA stability by inhibiting the RNA-binding protein (RBP) quaking (QKI) expression, a protein family that belongs to the signal transduction and activation of RNA (STAR) family and plays a crucial role in post-transcriptional gene regulation by binding to specific RNA sequences to influence the splicing, stability, localization, and translation of target mRNAs [29]. QKI is particularly important for cell differentiation, myelination, and cardiovascular development, with key functions in the brain, heart, and vascular systems.

### 2.2. Oxidative Stress

Oxidative stress is caused by an imbalance between the generation of ROS in tissue and a deficit of antioxidants, as found in DOX-treated cancer patients [30]. DOX possesses a high affinity for myocardial tissue and mitochondrial cardiolipin, thus interacting with mitochondrial DNA, with the inhibition of the respiratory chain [31]. This event leads to ROS generation, with the alteration of the phospholipids of cell membranes, mitochondria and the endoplasmic reticulum (ER), ultimately provoking myocardial cell damage [32].

Furthermore, DOX reduces the levels of glutathione and catalase (CAT), which act as antioxidants, thus aggravating oxidative stress [33]. In detail, DOX exhibits a quinone moiety, thus behaving as an electron acceptor. NADPH oxidase converts DOX into a semiquinone, which in turn reacts with oxygen, forming superoxide, with the contemporary regeneration of the quinone form. Superoxide interacts with nitric oxide (NO) to form the peroxynitrite anion, which is converted into peroxide by superoxide dismutase (SOD). In conclusion, the accumulation of hydroxyl radicals leads to DNA, protein and lipid damage in the context of myocardial cells [34,35,36].

The DOX-induced generation of ROS and reactive nitrogen species (RNS) affects endothelial cell function. In fact, DOX-mediated cardiomyopathy is characterized by increased levels of endothelin-1, activation of the type A and type B receptors, followed by vasoconstriction, and release of NO, adrenomedullin, and prostacyclin [37]. In this framework, it is worth discussing the role of the Yes-associated protein (YAP), which plays a crucial role in cell biogenesis, inducing the CAT and SOD transcriptions [38]. Moreover, in vivo studies have demonstrated that DOX-based therapy is associated with a reduction of the intramyocardial YAP 1 protein, as well as of target genes, such as CTGF, Birc5, and PARK2 [39,40]. Conversely, YAP1 overexpression abrogated DOX-induced cell death, inhibiting caspase-3/7 [40].

### 2.3. Autophagy

Autophagy consists of altered proteins or organelles, which are enclosed within double-membrane autophagosomes and then transported to lysosomes or vacuoles for degradation and further recycling [41]. Evidence has been provided that autophagy plays a crucial role in maintaining cardiac homeostasis and function, and therefore its dysregulation may cause DIC [42]. In this respect, DOX increases the levels of beclin-1, p62, and the microtubule-associated protein 1A/1B light chain 3 (LC3)-II/LC3-I [43]. Damaged mitochondria are removed through the mitophagy initiation driven by the activation of the PTEN-induced kinase and parkin [43]. Also, there is evidence that DOX alters mitochondrial function by decreasing the mitochondrial membrane potential, with the impairment of oxidative phosphorylation and its biosynthetic ability [44]. Furthermore, DOX abrogates lysosome biosynthesis and catepsin activity, with autophagolysosomal accumulation and the impairment of autophagy [45]. Fusion between autophagosomes and lysosomes promotes autophagic flux, while DOX inhibits the mechanism of fusion by enhancing STAT3 phosphorylation, upregulating lipocailin-2, and abolishing the interaction between syntaxin17 and the vesicle-associated membrane protein 8 [46]. Furthermore, DOX enhances the Toll-like receptor (TLR)-9 expression, inhibiting p38 MAPK, autophagy, while increasing ROS levels. There is evidence that the DOX-induced suppression of the AMPK activation and autophagy can aggravate apoptosis [47]. These events occur in the presence of activated PTEN-induced kinase 1 and parkin. Notably, the use of the mitophagy inhibitor mdivi-1 protects against DIC, preserving the mitochondrial membrane potential and the peroxisome proliferator-activated receptor gamma coactivator 1-alpha [36].

### 2.4. Inflammation

There is evidence that DIC induces cardiac inflammation via the release of an array of cytokines, which in turn aggravate the myocardial damage [48]. DOX treatment provokes ER stress and enhances mitochondrial iNOS levels in vivo, with the activation of the NF-kB pathway and generation of pro-inflammatory cytokines, e.g., interleukin-6 (IL-6) and IL-8 [49]. Moreover, DOX activates the TLR-4 in myocardial cells, with subsequent upregulation of the NF-kB pathway [50]. Furthermore, DOX generates the TLR2–MyD88 complex, with the activation of NF-kB and release of the tumor necrosis factor-alpha, IL-1 beta, IL-6, IL-8, IL-12, and IL-17, thus contributing to the cardiac inflammatory process and fibrosis [51]. In addition to the above-mentioned pathways, DOX treatment activates the NOD-like receptor thermal protein domain associated protein 3 (NLRP3) inflammasome, with caspase-1 activation, secretion of IL-1 beta and induction of pyroptosis, which triggers acute myocardial inflammation [52]. Pyroptosis is a form of pro-inflammatory cell death based on the swelling and plasma membrane rupture caused by the cleavage of gasodermin D, thus leading to the release of IL-1 beta, IL-18 and inflammatory cardiac damage [53]. DOX-induced pyroptosis increases the expression of the Bcl-2/adenovirus E1B interacting protein in mitochondria, with the activation of caspase-3 [54].

DOX treatment has an impact on cardiac smooth muscle cells, which switch from a contractile state toward a synthetic state, also differentiating into macrophage-like cells, which migrate to the intima [55]. These events contribute to vascular wall low-grade inflammation, with mucoid and fibrinoid swelling, necrosis, sclerosis, and hyalinosis, thus leading to further stiffness of the vascular wall. In addition, DOX impairs sarcoplasmic reticulum Ca^2+^ uptake by binding to cardiac ryanodine receptors and sarco/endoplasmic Ca^2+^ ATPase [56].

The role of macrophages in DIC has recently been investigated, especially considering the role played by the cardiac immune response in cardiovascular disorders [57,58]. In DOX-treated mice, macrophage infiltration was antecedent to cardiomyocyte damage. Mechanistically, the release of catecholamines by macrophages leads to mitochondrial apoptosis of cardiomyocytes through beta-AR stimulation, while macrophage depletion prevents cardiac damage.

### 2.5. Apoptosis and Ferroptosis

DIC-induced apoptosis depends on the modulation of the apoptosis-related genes exerted by DOX [59]. In this respect, in DOX-treated cardiomyocytes, the expression of the anti-apoptotic protein, Bcl-2, decreases paralleled with the increase in the Bcl-2-associated X expression, thus leading to the increased mitochondrial membrane permeability, cytochrome c release, and caspase-3 expression, respectively, which generates cardiomyocyte apoptosis [60].

Ferroptosis is a form of apoptosis, which depends on iron and is mediated by lipid peroxidation [61]. Cardiomyocytes treated with DOX undergo ferroptosis due to glutathione peroxidase 4 (GPx4) inactivation [62]. In fact, ferroptosis inhibitors, e.g., Fer-1, upregulate GPx4, with the reduction of Fe^2+^ accumulation and the mitigation of myocardial damage [63]. In this regard, DOX causes the accumulation of heme oxygenase 1 that can degrade heme [64]. On the other hand, the DOX-mediated methyltransferase-like 14 overexpression upregulates the transferrin receptors and, consequently, the uptake of iron, regulating the KCNQ1OT1–miR-7-5p–TFRC axis [65]. Additionally, DOX induces ferroptosis by the impairment of autophagy through the forkhead box 04 (FOXO4) transcription, and the high-mobility group box 1 nuclear translocation [66]. The major pathogenetic mechanisms of DIC are indicated in Table 1.

## 3. Role of Sirtuins in DIC

Sirtuins (SIRT) belong to the class-II-type histone deacetylase family, encompassing seven members from SIRT 1 to SIRT7. They modulate tissue metabolism, oxidative stress, and apoptosis through the deacetylation of target proteins, thus influencing cardiovascular function [67]. Evidence has been provided about their regulatory role in the onset of myocardial hypertrophy, ischemia/reperfusion injury, as well as diabetic cardiomyopathy [68]. On these grounds, the potential role of SIRT1–7 in DIC has been explored. In this respect, it has been documented that the SIRT 1 expression is reduced in in vitro and in vivo models of DIC, while its moderate overexpression protects cardiomyocytes from oxidative stress and apoptosis [69]. Moreover, resveratrol (RES), activating SIRT 1, abrogates the mitochondrial dysfunction and oxidative stress exerted by DOX [70].

SIRT 1 inhibits ROS generation, regulating the expression of CAT and manganese (Mn) SOD, deacetylating FOXO1, and thus decreasing cardiac oxidative stress [71]. Furthermore, SIRT 1 deacetylates and activates the peroxisome proliferator-activated receptor gamma coactivator 1 alpha, as well as NF-kB, thus improving myocardial oxidative stress and cardiac remodeling [72]. Also, the inhibition of PARP-2 increases the expression of SIRT 1, alleviating DIC [73].

A series of studies demonstrated that SIRT 1 activation can mitigate DOX-mediated cardiac inflammation in vitro, inhibiting NF-kB activation. For instance, the fibroblast growth factor 21 increases the activation of the SIRT 1/liver kinase B1/AMPK pathway, thus blocking NF-kB p65, with abrogation of the high expression of TNF-alpha and IL-6 [74]. Targeting the paternally expressed gene 3 upregulates SIRT 1 by the inhibition of miR-200a-3p, thus leading to the inhibition of NF-kB and the improvement of DOX-induced inflammation [75]. Some natural compounds can modulate SIRT1 activity. For instance, Jaceosidin inhibits the phosphorylation of IKKbeta and the translocation of NF-kB via SIRT 1 activation, ultimately suppressing NF-kB, while improving DOX-induced inflammation [62]. Of note, Jaceosidin does not impair DOX’s antitumor ability. Other natural compounds, such as calycosin and dihydromyrecitin, abrogate NLRP3 inflammasome, thus mitigating DOX-induced cardiac inflammation [76,77].

As far as DOX-mediated apoptosis is concerned, RES activates SIRT 1, attenuating the acetylation of the p53 protein, and thus alleviating DOX-mediated myocardial cell apoptosis [78]. Also, ferroptosis is reduced by the SIRT 1 activation via the Nrf2/Kelch-like-associated protein, with the mitigation of DIC [79]. SIRT 2 is present in cytoplasm as well as in mitochondria, where it regulates autophagy and mitophagy. For instance, SIRT 2 activates FOXO3a, with the upregulation of manganese superoxide dismutase (MnSOD) and attenuation of the ROS generation by cardiomyocytes [80]. Also, the AMPK modulation by SIRT 2 improves age-related cardiac dysfunction, thus mitigating DIC [81]. Also, the regulation of the SIRT 2/NRF2 pathway through the inhibition of miR-140-5p reduces DOX-induced antioxidative stress [82]. SIRT 3 is located within the mitochondria and its absence causes mitochondrial dysfunction, which results in oxidative stress, apoptosis, and inflammation, as observed in cardiac diseases [83]. Experimentally, the activation of SIRT 3 with RES, daidzein, and tubeimoside could alleviate DOX-induced myocardial inflammation [70,84]. Furthermore, the regulation of SIRT 3 by berberine could suppress DOX-induced cardiac oxidative stress [85]. Also, dichloroacetic acid upregulates the PGC-1 alpha/SIRT 3 pathway, improving DOX-induced mitochondrial dysfunction, oxidative stress, and apoptosis [86]. Furthermore, the Chinese herbal medicine, Qishen granules, increased the SIRT 3 expression, with decreased mitochondrial ROS generation and protection against DIC [87]. With special reference to autophagy, the SIRT 3 overexpression could inhibit miR-34-5p, leading to anti-autophagic activity and protection from DIC [88]. In addition, evidence has been reported that SIRT 3 inhibited the NLRP3 inflammasome, regulating autophagy and mitigating pyroptosis [76].

SIRT 4 is implicated in the regulation of myocardial energy metabolism, but its activity in cardiovascular diseases remains controversial. In fact, it inhibits fatty acid oxidation in muscles, suppressing malonyl-CoA decarboxylase, as well as SIRT 3-mediated MnSOD activity, thus affecting mitochondrial function [89]. Conversely, the overexpression of SIRT 4, as well as its interaction with optic atrophin 1, leads to the inhibition of autophagy and ROS generation [90,91]. With special reference to DIC, it has been documented that the overexpression of SIRT 4 activates the akt/mTOR pathway, thus inhibiting DIC [92].

SIRT 5 is a mitochondrial sirtuin, which plays a role in keeping mitochondrial homeostasis. To the best of our knowledge, one publication has investigated the role of SIRT 5 in DIC, following its overexpression by coenzyme Q 10, with protection from DOX-induced cardiac oxidative stress in mice [93].

SIRT 6 is a nuclear sirtuin, which regulates aging, oxidative stress, inflammation, and autophagy [94]. The SIRT 6 overexpression caused by targeting miR-330-5p inhibits ROS generation, as well as the apoptosis and necrosis induced by the p53/Fas pathway, ultimately leading to the mitigation of oxidative stress and cardiac atrophy related to DOX treatment [80,95]. Furthermore, SIRT 6 enhances autophagy by deacetylating and inhibiting SKG1, thus reducing DIC on the one hand, and on the other hand, augmenting the therapeutic efficacy of DOX through metabolic remodeling [96]. In addition, very recent evidence has documented that SIRT 6 can activate the proliferator-activated receptor alpha, abrogating the myocardial aging induced by DOX [97].

SIRT 7 is a nuclear sirtuin acting on the heart, liver, and spleen. Studies have demonstrated that SIRT 7 reduces myocardial stress via the deacetylation of p53, regulates autophagy through TGF beta, mitigates cardiac hypertrophy through the deacetylation of GATA4 and decreases cardiac hypoxia and apoptosis through the inhibition of miR-148-3p, respectively [98,99,100,101,102].

Sirtuin agonists have been investigated for possible mitigation of DIC. There is evidence that RES can act as a natural agonist of SIRT 1, even if the mechanism of action is still controversial. A report has documented that RES enhances the activity of SIRT 1 via binding to lamin A [103]. Another study has reported that RES molecules interact with the fluorescent moiety of the substrate p53 peptide and the N-terminal regulatory region of SIRT 1, thus enhancing its activity [104].

Other natural products, such as dihydrocoumarin, berberine, and limonin, improve DOX-induced cardiotoxicity, but they exhibit poor bioavailability and low specificity even when encapsulated into liposomes [105]. Furthermore, synthetic SIRT 1 agonists, e.g., SRT1720 have been tested on cardiomyocytes, with the induction of autophagy through the SIRT1/AMPK pathway and protection against hypotoxic stress [106]. However, no evidence of the effects of SRT1720 on DIC has been reported. Parallelly, a few studies have been focused on the effects of sirtuin inhibitors, such as EX527 and 3-TYP, on experimental DIC. Both inhibitors can abrogate the protective effects of RES, berberine, and ononin against DOX-induced cardiotoxicity [107]. The main effects of sirtuins on DIC are illustrated in Table 2.

## 4. Role of Natural Products in DIC

Evidence has been provided that natural products, such as polyphenols, are endowed with antioxidant and anti-inflammatory activities, neutralizing certain pathways, e.g., NF-kB and NLRP3 inflammasomes involved in mechanisms of various tissue damage [111,112,113,114].

RES in combination with the fibroblast growth factor 1 can ameliorate murine DIC via the activation of the SIRT1-NRF2 pathway [115], as well as ferroptosis acting on the MAPK pathway [109]. Quercetin can abolish ROS production in DIC, preventing the opening of the mitochondrial permeability transition pore [116]. Also, rutin and apigenin have been shown to reduce DOX-induced apoptosis and autophagy [117,118]. Allicin has been shown to improve DIC in rats, suppressing oxidative stress, inflammation, and apoptosis [48]. A series of compounds, e.g., neferine, astragaloside IV, acacia hydaspica, and resolvin D1 have been reported to reduce DIC, suppressing NADH oxidase activity [119,120,121,122]. Evidence has been provided that show Panax ginseng mitigated DIC, upregulating the Nrf2–ARE axis, with a reduction of ROS production [123].

### Therapeutic Attempts to Reduce DIC

The encapsulation of DOX in nanostructures has been applied to decrease DIC. Doxil, a pegylated liposomal system into which DOX has been incorporated, has been shown to reduce DIC [124]. In breast cancer patients, liposomal DOX-based chemotherapy was more advantageous in terms of progression-free survival and DIC reduction than conventional DOX [125]. Also, magnetic iron oxide nanoparticles are effective nanocarriers for DOX therapy, but they are not yet in clinical trials [126]. The iron chelator dexrazoxane can bind iron, preventing its entry into cardiomyocytes, thus reducing cardiac damage [127]. Its cardioprotective mechanism involves two key actions: (1) it binds free iron and consequently reduces the ROS formation generated by doxorubicin–iron complexes, thereby minimizing the oxidative damage to cardiomyocytes, and (2) it selectively inhibits topoisomerase IIβ in the heart, preventing doxorubicin-induced DNA double-strand breaks without interfering with the drug’s anticancer efficacy (which primarily depends on topoisomerase IIα inhibition in tumor cells) [128]. Clinical studies have demonstrated that dexrazoxane significantly reduces the risk of cardiomyopathy and heart failure in patients receiving high-dose or long-term doxorubicin therapy, particularly in pediatric and adult oncology populations [129,130]. While its use was initially restricted due to theoretical concerns about secondary malignancies (largely unsubstantiated in long-term follow-ups), the current guidelines support its use in high-risk patients to preserve cardiac function without compromising chemotherapy outcomes [131,132]. Overall, while dexrazoxane is highly effective in preventing doxorubicin-induced cardiotoxicity, its use requires careful patient selection and monitoring to manage the potential adverse effects. Ongoing research is exploring optimized dosing strategies and novel derivatives to further enhance its protective effects. Carvedilol, a vasodilator and beta adrenoceptor antagonist, is endowed with antioxidant effects, thus reducing DIC [133]. The statin rosuvastatin has demonstrated cardioprotective effects in mice treated with DOX, but clinical trials have generated controversial results [134,135].

The RNA-binding protein (RBP) seems to be another promising target to regulate cardiac pathophysiology: it was recently reported that Qki 5 overexpression significantly reduces doxorubicin-induced toxicity by modulating the biogenesis and function of cardioprotective circular RNAs, which help maintain cellular homeostasis and mitigate DNA damage and oxidative stress [136].

Quite interestingly, a very recent report has documented that inhibition of neutrophil extracellular traps (NETs) prevents DIC in mice [137]. In detail, NETs prime macrophages for IL18 release in DOX-treated-mice with the production of interferon-gamma and the tumor necrosis factor-alpha from activated T cells. Both cytokines downregulate the expression of Cx43, provoking cardiac conduction disorders. In reference to the above concept, the accumulation of NETs has been found in the blood of DOX-treated cancer patients [138]. Therefore, targeting NETs may represent a new therapeutic strategy in human DIC.

## 5. Conclusions

DOX treatment exhibits severe cardiac side effects, including arrhythmia, heart failure, and ventricular dysfunction. Oxidative stress, DNA instability, inflammation, autophagy, and apoptosis are the major causes of DIC.

Understanding the balance between its cytotoxic and adverse effects remains crucial to optimizing its therapeutic outcomes in cancer treatment. A better clarification of the mechanism responsible for DIC may help in the identification of drugs able to prevent or alleviate DIC. Dexrazoxane is the only drug approved by the FDA; however, some side effects have been reported after clinical use. Other strategies based on DOX nanoformulations or administration of natural compounds have demonstrated their efficacy only in pre-clinical studies. Therefore, this area of investigation needs to be more explored to discover more appropriate compounds for treating DIC.

## Figures and Tables

**Table 1:** Pathogenesis of DIC.

Oxidative Stress	Interaction with mitochondrial DNA, and inhibition of the respiratory chain [31];
	Generation of ROS, alteration of the phospholipids of cell membranes, mitochondria, and ER, with myocardial damage [32];
	Reduction of glutathione and CAT levels, with increase in oxidative stress [33]; DIC-mediated endothelial dysfunction with increased levels of endothelin-1, activation of type A and type B receptors, vasoconstriction, and release of NO, adrenomedullin, and prostacyclin [37]
Autophagy	DOX-mediated increase in Beclin-1, p62, and microtubule-associated protein 1A/1B light chain 3 (LC3)-II/LC3-I levels, with damaged mitochondria removed by mitophagy [43];
	DOX-mediated abrogation of lysosome biosynthesis and catepsin activity, with autophagolysosomal accumulation, and impairment of autophagy [45]; DOX-induced inhibition of the fusion between autophagosomes and liposomes [46]; DOX-mediated enhancement of TLR9, and suppression of AMPK activation [47]
Inflammation	DIC-induced release of pro-inflammatory cytokines through activation of TLR-4, NF-kB, and NLRP3 inflammasome [48,49,50];
	DOX-mediated transformation of smooth cardiac muscle cells into macrophage-like cells, with vascular wall low-grade inflammation and impairment of sarcoplasmic reticulum [55,56];
	DOX-induced apoptosis of cardiomyocytes via release of catecholamines by infiltrating macrophages [57]
Apoptosis/Ferroptosis	Decrease in the anti-apoptotic protein, Bcl-2, and increase in Bcl-2-associated X expression in DOX-treated cardiomyocytes, with increased mitochondrial permeability, cytochrome c release and caspase-3, thus leading to cardiomyocyte apoptosis [60];
	DOX-induced ferroptosis of cardiomyocytes by inactivation of GPx4 [62,63,64]; Overexpression of methyltransferase-like 14 with upregulation of transferrin receptors, and uptake of iron [65]; DOX-mediated ferroptosis by impairment of Forkhead Box O4 transcription, and high mobility group Box 1 nuclear translocation [66]

**Table 2:** Beneficial effects of sirtuins on DIC.

SIRT 1	ROS generation and FOXO 1 deacetylation inhibition, with decrease in cardiac oxidative stress [71]
	SIRT 1-mediated inhibition of NF-kB through deacetylation of the peroxisome proliferator-activated receptor gamma coactivator 1 alpha; SIRT 1/Liver kinase B1/AMPK pathway activation; inhibition of miR-200a 3p, respectively [72,75,79]
	Natural product (jaceosidin, calycosin, and dihydromyrecitin)-mediated activation of SIRT 1, with inhibition of NF-kB [76,77,94]; p53 protein acetylation decrease by resveratrol-activated SIRT 1, with attenuation of myocardial cell apoptosis [78]; SIRT 1-mediated activation of the Nrf2/Kelch-like associated protein, with reduction of ferroptosis, and DIC [79]
SIRT 2	FOXO3a and AMPK SIRT 2-mediated activation, respectively, with reduced release of ROS and mitigation of DIC [81,108]
	SIRT 2 activation and improvement of DOX-mediated cardiac aging [65]; miR-140-5p inhibition with activation of the SIRT2/NRF2 antioxidant pathway [82]
SIRT 3	Natural product (RES, daidzein, tubeimoside, berberine, Qishen granules) and dichloroacetic acid-mediated upregulation of SIRT3, with improvement of DOX-induced mitochondrial dysfunction, ROS generation, and apoptosis [70,84,85,86,87,109]
	miR-34-5p SIRT 3-mediated inhibition, with autophagic activity regulation, and protection from DIC [88]
	SIRT 3-induced inhibition of NLRP3 inflammasome, with autophagy regulation, and pyroptosis decrease [83]
SIRT 4	SIRT 4-mediated inhibition of fatty acid oxidation in muscles, with reduction of mitochondrial function [89]
	Overexpression of SIRT 4 and interaction with optic atrophin 1, with regulation of autophagy and ROS generation; SIRT 4-mediated inhibition of DIC, with activation of the akt/mTOR pathway [92]
SIRT 5	Coenzyme Q 10-mediated overexpression of SIRT 5 and protection from DIC [93]
SIRT 6	SIRT 6 overexpression by targeting miR-330-5p, with inhibition of ROS generation, apoptosis, and necrosis during DIC [80,95]
	Enhancement of autophagy by SIRT 6-mediated acetylation and inhibition of SKG1 [110]
SIRT 7	SIRT 7-induced reduction of myocardial stress via deacetylation of p53 and GATA4; regulation of
	autophagy and inhibition of miR-148-3p, respectively [83,98,100,101,102]

## Data Availability

The authors confirm that the data supporting the findings of this study are available within the article.

## Abbreviations

The following abbreviations are used in this manuscript:

CATI	Catalase
DICJ	Doxorubicin-induced cardiotoxicity
DOX	Doxorubicin
ER	Endoplasmic reticulum
GPx4	Glutathione peroxidase 4
IL	Interleukin
MnSOD	Manganese superoxide dismutase
NLRP3	Nod-like receptor thermal protein domain-associated protein 3
NO	Nitric oxide
NOX	NADH oxidase
RBP	RNA-binding protein
RES	Resveratrol
ROS	Reactive oxygen species
SIRT	Sirtuins
TLR	Toll-like receptor
YAP	Yes-associated protein
